# Comparative genomics *Lactobacillus reuteri* from sourdough reveals adaptation of an intestinal symbiont to food fermentations

**DOI:** 10.1038/srep18234

**Published:** 2015-12-11

**Authors:** Jinshui Zheng, Xin Zhao, Xiaoxi B. Lin, Michael Gänzle

**Affiliations:** 1 Dept. of Agricultural, Food, and Nutritional Science, University of Alberta, Edmonton, Canada; 2 State Key Lab of Agricultural Microbiology, Huazhong Agricultural University, Wuhan, China; 3 School of Food and Pharmaceutical Engineering, Hubei University of Technology, Wuhan, P.R. China

## Abstract

*Lactobacillus reuteri* is a dominant member of intestinal microbiota of vertebrates, and occurs in food fermentations. The stable presence of *L. reuteri* in sourdough provides the opportunity to study the adaptation of vertebrate symbionts to an extra-intestinal habitat. This study evaluated this adaptation by comparative genomics of 16 strains of *L. reuteri*. A core genome phylogenetic tree grouped *L. reuteri* into 5 clusters corresponding to the host-adapted lineages. The topology of a gene content tree, which includes accessory genes, differed from the core genome phylogenetic tree, suggesting that the differentiation of *L. reuteri* is shaped by gene loss or acquisition. About 10% of the core genome (124 core genes) were under positive selection. In lineage III sourdough isolates, 177 genes were under positive selection, mainly related to energy conversion and carbohydrate metabolism. The analysis of the competitiveness of *L. reuteri* in sourdough revealed that the competitivess of sourdough isolates was equal or higher when compared to rodent isolates. This study provides new insights into the adaptation of *L. reuteri* to food and intestinal habitats, suggesting that these two habitats exert different selective pressure related to growth rate and energy (carbohydrate) metabolism.

*Lactobacillus reuteri* persist in intestinal microbiota of vertebrate animals as well as in food fermentations^1^,^2^,^3^,^4^. *L. reuteri* colonizes humans and animal hosts^2^,^4^; the phylogenetic differentiation of strains of *L. reuteri* originating from different hosts reflects co-evolution of *L. reuteri* with its vertebrate hosts^4^. This evolutionary adaptation differentiates the species *L. reuteri* in host-adapted phylogenetic lineages comprised of isolates from rodents (lineages I and III), humans (lineages II and VI), pigs (lineages IV and V), and poultry (lineage VI)^4^,^5^.

*L. reuteri* also occur in industrial sourdoughs^6^ and cereal fermentations in tropical climates^1^,^7^. Sourdoughs are typically maintained by continuous propagation, a process which rapidly selects for the most competitive microbiota. Major selection criteria for fermentation microbiota in cereal ecosystems are rapid growth in cereal substrates, and acid resistance^1^,^8^,^9^,^10^. Food isolates of *L. reuteri* match to host-adapted lineages^11^ and maintain host-specific physiological traits^12^,^13^,^14^, including the ability to colonize the lineage-specific hosts^11^,^15^.

The differentiation of *L. reuteri* into host-adapted lineages implies that an extra-intestinal habitat did not exist for a majority of the evolution of this species^14^. However, the occurrence of *L. reuteri* in the human-made habitat sourdough provides the opportunity to study the “reverse adaptation” of vertebrate symbionts to an extra-intestinal habitat. This study employed comparative genomics of *L. reuteri* to evaluate the genetic determinants of this adaptation or selection process. Genome sequences of intestinal strains of *L. reuteri* were retrieved from public databases and compared to four genome sequences of rodent-lineage sourdough isolates^16^. The sourdough isolates *L. reuteri* LTH2584, TMW1.112 and TMW1.656 originate from SER sourdough, a sourdough that is used industrially for production of a baking improver^9^. This sourdough has been maintained by continuous propagation since about 1970. *L. reuteri* LTH2584, TMW1.1112 and TMW1.656 were isolated from this sourdough in 1988, 1994, and 1998^6^,^9^; all of these strains produce reutericyclin, a tetramic acid derivative with antimicrobial activity against Gram-positive bacteria^6^,^16^. *L. reuteri* LTH5448 was isolated from a different sourdough processed at the same facility in 2000^8^,^17^; this strains does not produce reutericyclin but maintains the reutericyclin genomic island and reutericyclin resistance^16^,^17^. Comparative genomics analyses included analyses of the core genome as well as gene gain and gene loss events that were studied on the basis of the pan-genome. We also performed positive selection analysis for these core genes of the whole species. Finally, the competitiveness of sourdough isolates of *L. reuteri* in model sourdoughs was compared to the competitiveness of closely related intestinal isolates.

## Results

### Phylogenetic analysis of 16 sequenced *L. reuteri* strains including 4 sourdough strains

**Figure 1:**
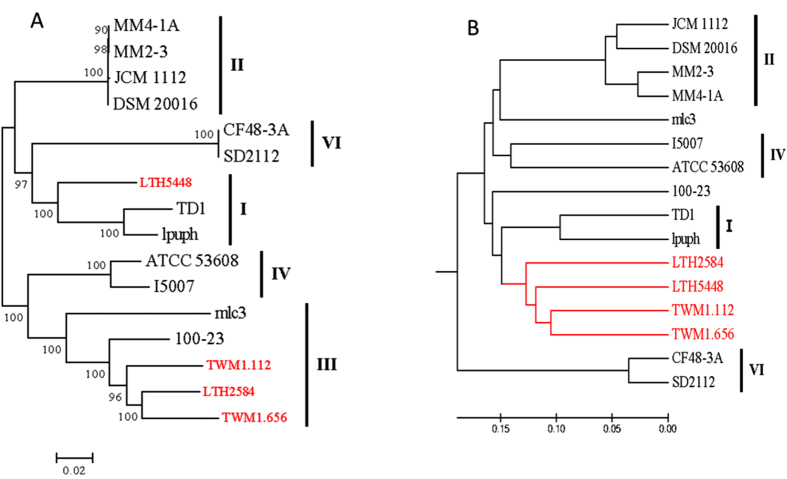
Phylogenetic analysis of the 16 *L. reuteri* strains. (**A**) Phylogenetic tree based on core genes. Roman numerals designate the host adapted lineages of *L. reuteri*^4^,^15^. Strains isolated from sourdough are marked in red. Only bootstrap values above 90 were shown. Branch lengths are proportional to the number of substitutions per site (see scale bar). (**B**) Genome tree based on gene content matrix. Scale bar represent the genetic distance as determined by Jaccard distance.

The phylogenetic analysis was carried out with all available genome sequences of *L. reuteri*, including 4 genome sequences of sourdough isolates^16^. A phylogenetic tree was constructed based on the core genome of *L. reuteri* (Fig. 1A). Strains of *L. reuteri* were grouped into 5 clusters corresponding to the host-adapted lineages I (rodent), II (human), III (rodent), IV (pig) and VI (poultry and human). Sourdough strains were assigned to the rodent-adapted lineages I and III, in agreement with previous analyses^11^. *L. reuteri* LTH5448 clustered with lineage I rodent isolates; *L. reuteri* LTH2584, TWM1.112 and TWM1.656 were grouped into lineage III together with the rodent isolates *L. reuteri* 100-23 and mlc3. *L. reuteri* LTH2584, an SER sourdough isolate obtained in 1988, was more closely related to *L. reuteri* TWM1.656, which was isolated from SER sourdough in 1998, than to *L. reuteri* TWM1.112, which was isolated from the same sourdough in 1994^6^.

A gene content tree was constructed to study the gain and loss of genes among these strains. Here, strains sharing more genes were clustered together (Fig. 1B). The topology of the gene content tree was different from the core genome phylogenetic tree, indicating gene loss or acquisition of genes by horizontal genetic transfer. Three clusters corresponding to linages II, IV and VI were maintained but the gene content tree highlighted differences between strains in each cluster. For example, the four lineage II *L. reuteri* MM4-1A, MM2-3, DSM 20016 and JCM 1112 were not separated in the core genome phylogenetic tree but differentiated in two groups by calculating the gene content tree (Fig. 1B). *L. reuteri* DSM20016 and JCM1112 were derived from the same original isolate, F275, and differences between these two strains may reflect loss of genes during propagation in the laboratory^18^. The two lineage III strains *L. reuteri* 100-23 and mlc3 showed a quite different gene content. Remarkably, all four sourdough isolates were grouped together despite their divergent phylogenetic origin. *L. reuteri* LTH5448 was more closely related to *L. reuteri* LTH2584 than to *L. reuteri* TWM1.112 and TWM1.656.

### Comparative analysis of sourdough strains

**Figure 2:**
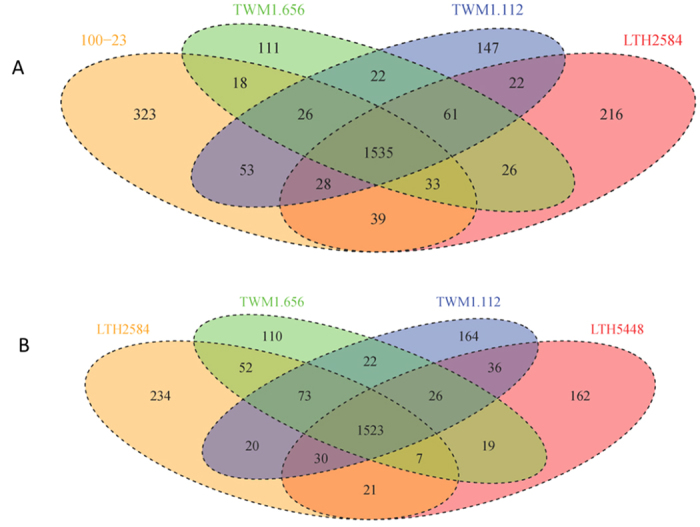
Comparative analyses of genes shared between sourdough isolates of *L. reuteri* and *L. reuteri* 100-23, and among sourdough isolates. (**A**) Venn diagram of core, distributed and unique gene numbers between the lineage III strains *L. reuteri* LTH2584, TMW1.656, TMW1.112, and 100-23. (**B**) Venn diagram of core, distributed and unique gene numbers among the Lineage III sourdough isolates *L. reuteri* LTH2584, TMW1.656, TMW1.112, and the Lineage I sourdough isolate *L. reuteri* LTH5448.

To understand how the intestinal strains adapted to sourdough, and to identify genes that are unique to sourdough isolates, the gene content similarity and dissimilarity of these strains was analysed. *L. reuteri* LTH2584, TWM1.112, TWM1.656 and 100-23 shared 1535 core genes (Fig. 2A); this core genome is higher than the core genome of the whole species^5^, reflecting that all these strains are grouped in lineage I. *L. reuteri* LTH2584 and 100-23 had more unique genes than *L. reuteri* TWM1.112 and TWM1.656 (Fig. 2A), which contributed to the distinct position of the former two strains in the gene content tree. Sourdough isolates shared 1523 core genes (Fig. 2B). Genes that were shared by all sourdough isolates but absent in other strains include the chromosomally encoded reutericyclin genomic island^16^, a putative aspartate racemase, a LytTr-domain protein with putative regulatory function, and components of a putative ABC-transporter (Table 2). Genes that were only present in some of the sourdough isolates include a glycosyltransferases with putative function in protein glycosylation (LTH2584 and TMW1.656) and a putative hydroxyglutarate dehydrogenase (LTH2584 and TMW1.112) which catalyses the use of α-ketoglutarate as electron acceptor^19^. Of note, distributed genes that are present in sourdough isolates of *L. reuteri* and other strains include several putative enzymes of the shikimic acid pathway for biosynthesis of aromatic amino acids (Table 2). In summary, only genes coding for reutericyclin biosynthesis are unique to all sourdough isolates of *L. reuteri*.

### Positive selection of the core genes contributing to the adaptation of sourdough isolates

**Figure 3:**
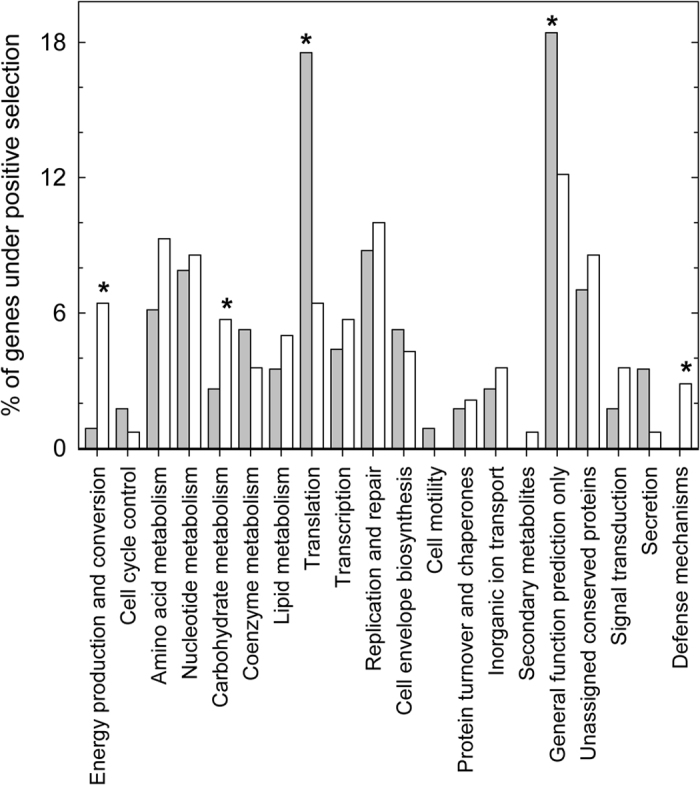
Proportions of positive selection of core genes in each COG category in all *L. reuteri* strains (gray bars), and the branch of sourdough isolates comprising *L. reuteri* LTH2584, TWM1.112, and TWM1.656 (white bars). Site model M2a and its null model M1a were compared to infer genes under positive selection in the whole species. Branch-site mode and its null model were compared to study genes under positive selection across the COG categories. COG categories that were significantly enriched in all strains of the species *L. reuteri*, or in the Lineage III sourdough isolates are marked by an asterisk.

Analysis of positive selection aimed to identify the selective pressure on the core genome of *L. reuteri*, and to determine whether sourdough and intestinal strains are subjected to a differential selective pressure. Initially, positive election was analysed in all 16 strains of *L. reuteri*. A total of 124 core genes were under positive selection (Fig. 3 and Table S2), representing 10.36% of the core genome. Among the genes that are under positive selection, 22% relate to metabolism, including transporters and enzymes for protein, amino acid, carbohydrate, and lipid conversion. Several genes under positive selection were listed as “general functional prediction only”, but most of these predicted functions were also related to metabolic functions. Other abundant genes under positive selection relate to DNA replication, recombination, and repair. When compared with the composition of the core genes, COG categories “translation, ribosomal structure and biogenesis” and “general functional prediction only” were significantly enriched among genes under positive selection in all 16 core genomes of *L. reuteri* (P = 0.04, 0.03, one-sided binomial test). For the 20 genes in the former category, 8 are tRNA associated genes, 6 are ribosomal protein genes, 3 are 23S RNA-specific pseudouridylate synthases, 2 are translation elongation factor genes, and 1 is methylase of polypeptide chain release factor gene (Table S2). For the latter category, most predicted functions relate to metabolism. For example, 6 out 21 were hydrolases, and other were some reductases, permeases and esterases.

To identify the selective pressure acting on the sourdough isolates, the branch-site model and its null model were used to compare the function categories under positive selection in the branch comprising *L. reuteri* LTH2584, TWM1.112, and TWM1.656 to all other strains in the species. A total of 177 core genes were under positive selection in these lineage III sourdough isolates (Fig. 3 and Table S3). Of these, 135 genes were under positive selection only in this branch and the remaining 42 were under positive selection in the sourdough isolates as well as the remainder of the species. Of the core genes under positive selection in the sourdough branch, 33% related to metabolism (Fig. 3 and Table S3). Three COG categories were significant enriched in the lineage III sourdough isolates, “Energy production and conversion” (P = 5.9 * 10^−6^), “Carbohydrate transport and metabolism” (P = 0.03) and “Defense mechanisms” (P < 2.2 * 10^−16^) (Fig. 3). Examples of gene in these COG categories that are under positive selection include key metabolic enzymes such as maltose phosphorylase, lactate dehydrogenase, alcohol dehydrogenase, and several sugar transport enzymes (Table S3).

### Competitiveness of *L. reuteri* strains in sourdough: experimental design

To determine whether genomic adaptation of *L. reuteri* to the sourdough environment increases the competitiveness of strains, competition experiments sourdoughs were carried out. Competition experiments in back-slopped sourdoughs are a sensitive tool to determine the competitiveness of strains because even small differences in competitiveness result in predominance of the more competitive strain after few refreshments^10^,^20^. Experiments were performed with fermentation cycles of 1, 2, or 3 days. The selection of strains used in the competition experiments included the sourdough isolates *L. reuteri* LTH5448, LTH2584, TMW1.112 and TMW1.656; and the rodent isolates *L. reuteri* 100-23, mlc3 and lpuph. Two methods were used to to achieve strain specific quantification of *L. reuteri* in sourdough. When used in combination, qPCR and differential plate counts ensured that sourdough microbiota in all samples consisted of only of those strains used as inoculum, and accurately quantified the strain specific contribution to the fermentation microbiota.

**Figure 4:**
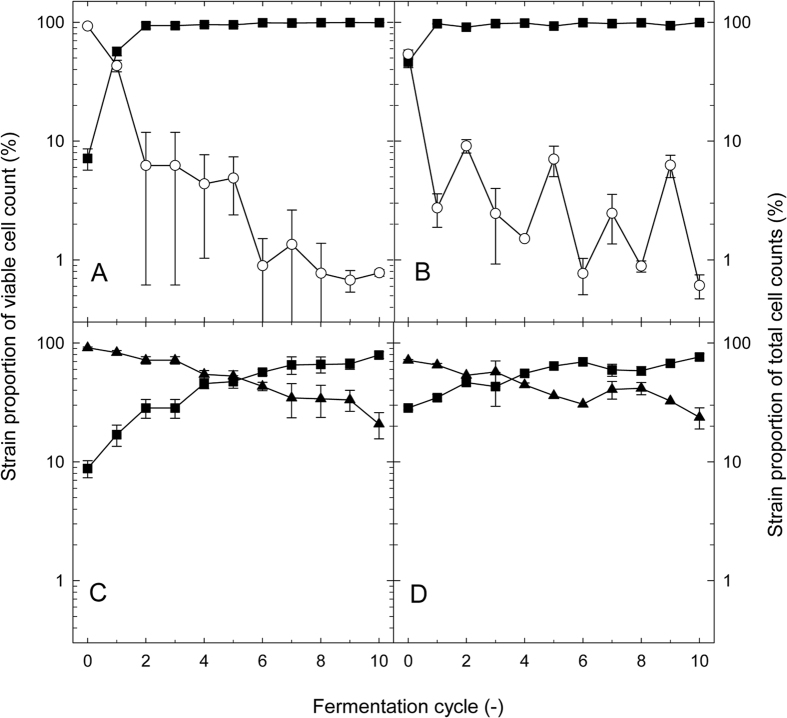
Composition of fermentation microbiota in binary strain competitions of strains of *L. reuteri.* Sourdoughs were inoculated with *L. reuteri* LTH2584 and 100-23 (Panels A and B) or with *L. reuteri* LTH2584 and LTH5448 (Panels C and D) and maintained by continuous back-slopping with 10% inoculum over 10 fermentation cycles with 1 d incubation times. Sourdough microbiota were analysed by differential plate counts (Panels A and C) and results were expressed as log proportion of the individual strains to the total viable cell counts. Sourdoughs were also analysed by qPCR targeting strain-specific sequences and log DNA copy numbers converted to cell counts using the strain-specific calibration curves (Panels B and D). Results were expressed as proportion of the individual strains to the total cell counts. Symbols represent *L. reuteri* strains LTH2584 (◼), LTH5448 (□) and 100-23 (◯).

The competition experiments were evaluated by calculation of the relative fitness. An example for the evaluation of competition experiments by qPCR and differential viable cell counts is shown in Fig. 4; all experiments are shown in Figure S3 of the online supplementary material. The cell counts and the pH of sourdough were influenced by the fermentation time and the fermentation microbiota. Three day fermentation cycles results in lower cell counts and higher pH values at the end of the fermentation when the glutamate-decarboxylase positive *L. reuteri* LTH5448 was part of the fermentation microbiota (Figure S4,S5).

### Competitiveness of *L. reuteri* strains in sourdough

**Figure 5:**
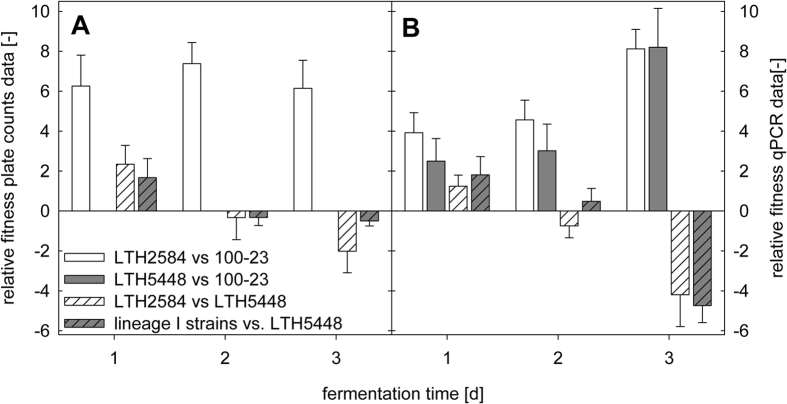
Relative fitness of strains of *L. reuteri* in sourdoughs backslopped with 1d, 2d, or 3d fermentation cycles. The relative fitness of strains was calculated from differential cell counts **(Panel A)** and from qPCR with strain specific primers **(Panel B)** by using the equation 
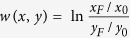
, where x, y denote the strains used in binary strain combinations, x_F_ and y_F_ denote the cell counts or gene copy numbers at the end of each fermentation cycle and x_0_ and y_0_ denote the cell counts or gene copy numbers at the beginning of each fermentation cycle. The relative fitness was calculated for the following strain combinations: *L. reuteri* LTH2584 versus 100-23 (white bars); *L. reuteri* LTH5448 versus 100-23 (grey bars); *L. reuteri* LTH2584 versus LTH5448 (white hatched); and lineage I strains consisting of *L. reuteri* LTH2584, TMW1.112 and TMW1.656 versus lineage III strain *L. reuteri* LTH5448 (grey hatched). Differential enumeration was not possible for the strain combination *L. reuteri* LTH5448 and 100-23, therefore, only qPCR data is shown for this strain combination. Each competition was performed in doughs with 10 fermentation cycles that were back-slopped every 1, 2 and 3 days per fermentation cycle, respectively. The data was expressed as mean ± standard deviation from experiments performed in duplicate with two technical replicates.

**Figure 6:**
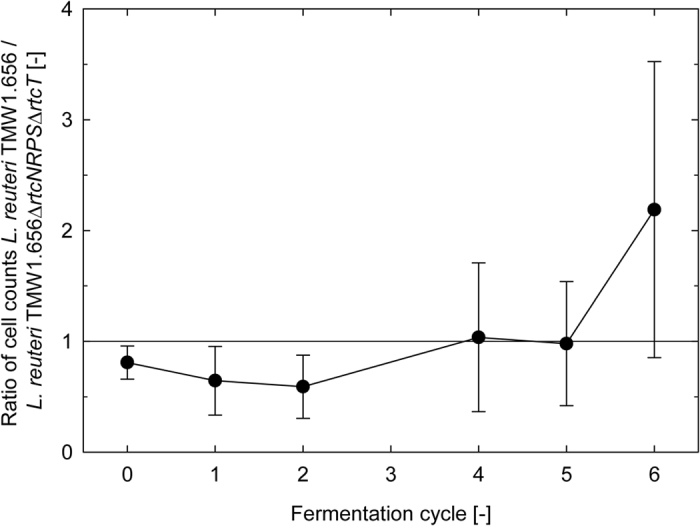
Composition of fermentation microbiota in sourdoughs inoculated with the reutericyclin producing wild type strain *L. reuteri* TMW1.656 and the isogenic reutericyclin-negative and reutericyclin-sensitive *L. reuteri* TMW1.656Δ*rtcN*Δ*rtcT*^16^. The ratio of wild type strain to mutant strain was determined by qPCR analysis with strain-specific primers. Results are shown as means ± standard deviation of triplicate independent experiments.

The competitiveness of the sourdough isolates *L. reuteri* LTH2584 and LTH5448 was substantially higher when compared to the rodent isolate *L. reuteri* 100-23 (Fig. 5). This difference in competitiveness to the reutericyclin-sensitive *L. reuteri* 100-23 was equivalent for the reutericyclin-producing *L. reuteri* LTH2584 and the reutericyclin-negative *L. reuteri* LTH5448. To determine whether this increased competitiveness was influenced by reutericyclin-production, a competition experiment was carried out between the reutericyclin-producing *L. reuteri* TMW1.656 and the isogenic reutericyclin-negative and -sensitive *L. reuteri* TMW1.656Δ*rtcN*Δ*rtcT*^16^. *L. reuteri* TMW1.656 and TMW1.656Δ*rtcN*Δ*rtcT* exhibited a comparable competitiveness and were maintained in approximately equal cell counts over 6 fermentation cycles (Fig. 6). This result demonstrates that reutericyclin production does not substantially influence the competitiveness of *L. reuteri* strains in sourdough.

Competition experiments between sourdough isolates of *L. reuteri* revealed that all strains that were used as inoculum were maintained over 10 fermentation cycles (Figure S3 of the online supplemental material). The competitiveness of the sourdough strains reflected the effects of lineage-specific metabolic traits on competitiveness in sourdough^10^,^20^. Glutamate decarboxylase mediates acid resistance; presence of this enzyme also increases competitiveness in sourdoughs with a long fermentation time^10^. Accordingly, the glutamate-decarboxylase-negative lineage I strains were more competitive in sourdoughs that were maintained by short fermentation cycles while the glutamate-decarboxylase positive lineage III strain *L. reuteri* LTH5448 was more competitive in sourdoughs that were maintained with 2 and 3 d cycles (Fig. 5).

## Discussion

This study employed comparative genomics to demonstrate that the evolution of *L. reuteri* is shaped by positive selection of the core genome in addition to the gain and loss of accessory genes. Moreover, the identification of core genes under positive selection and the analysis of competitiveness in sourdough demonstrate that sourdough isolates of *L. reuteri* have adapted to the new habitat, or have been selected from a distinct subset of rodent lineage strains. This study provides new insights into the adaptation of *L. reuteri* to food and intestinal habitats, suggesting that these two habitats exert different selective pressure related to growth rate and metabolism.

### Core genome phylogenetic analysis confirms host-specific lineages

Core genome phylogenetic analysis of the 16 strains of *L. reuteri* confirmed differentiation into host-specific lineages^11^. The rodent lineage III strains *L. reuteri* LTH2584, TWM1.112, and TWM1.656 were isolated form the same sourdough in 1988, 1994, and 1998, respectively. The phylogenetic relatedness of these strains suggested that the later isolates may be isolates of the same organism after 10 years of adaptation to sourdough fermentation^6^. However, successive contamination of the same sourdough with different strains of rodent origin is an alternative explanation for the isolation of highly related strains from the same sourdough.

### Role of reutericyclin production for competitiveness in sourdoughs

Reutericyclin production may contribute to competitiveness of *L. reuteri* in sourdough^3^,^6^. This study demonstrated that a reutericyclin sensitive derivative of *L. reuteri* TMW1.656 and the reutericyclin producing wild type strain exhibited comparable competitiveness in sourdough, indicating that the ecological advantage of reutericyclin is about equivalent to the cost of reutericyclin production^16^,^21^. The reutericyclin gene cluster was acquired by horizontal gene transfer by few Lineage I and Lineage III sourdough isolates of *L. reuteri*^6^,^16^ and is thus unlikely to represent a sourdough-specific metabolic trait.

### Evolution of the intestinal symbiont *L. reuteri* by horizontal gene transfer and positive selection

The evolution of pathogens is driven by gene loss, acquisition of genes by horizontal gene transfer, and by positive selection of the core genome^22^,^23^,^24^. The relative contribution of recombination and positive selection are highly dependent on the species and the ecosystem. Only few genes were reported to be under positive selective pressure in the pathogenic *Listeria monocytogenes*^24^ while up to 34% and 92%, respectively, of the core genome were positively selected in specific lineages of the host-adapted genera *Streptococcus* and *Campylobacter*^22^,^23^. The evolution of the host adapted gut symbiont *L. reuteri* was previously attributed to gene loss and acquisition of lineage-specific accessory genes^15^. The congruent clustering of *L. reuteri* strains in the phylogenetic tree and the gene content tree confirms a major role of the gain or loss of host-specific metabolic and genetic traits in the evolution of the species^14^,^15^. This study additionally demonstrates that positive selection of the core genome shapes the evolution of *L. reuteri*. The expression of ribosomal proteins, rRNA and other transcription factors is regulated by the bacterial growth rate^25^ and a high density of ribosomal genes relates to rapid growth of *L. sanfranciscensis* in sourdough^26^. Positive selection in the functional category translation indicates that the intestinal ecological niches harbouring *L. reuteri* exert selective pressure for rapid growth. The proportion of the core genome that was under positive selection in *L. reuteri* matches the corresponding proportion in *S. mutans*^23^. *S. mutans* and *L. reuteri* are phylogenetically related host-adapted organisms colonizing the upper intestinal tract but contrast with respect to their impact on the hosts. *S. mutant* is a pathogen and *L. reuteri* is considered as probiotic but both species apparently use comparable ecological strategies for colonization and persistence^16^,^27^.

### Reverse evolution or selection of *L. reuteri* in an extra-intestinal habitat?

The persistence of *L. reuteri* over 10 years in sourdough fermentation provides a unique opportunity to study the adaptation or selection of a host-specific gut symbiont to an extra-intestinal environment. Several lines of evidence suggest that SER sourdough isolates are distinct from intestinal strains. First, sourdough isolates cluster separately in the gene content tree, indicating that horizontal gene transfer and the loss of genes relates to the transition to sourdough^16^. Second, the functional categories “energy production and conversion” and “carbohydrate metabolism”, which are key elements for competitiveness in sourdough^28^,^29^, were significantly enriched among the positively selected genes in SER isolates. Third, sourdough isolates of *L. reuteri* displayed a higher relative fitness in sourdough when compared to rodent isolates. *L. reuteri* LTH5448 also achieved a high proportion of cell counts in competition with rodent isolates *L. reuteri* mlc3 and lpuph in experiments with 2 d fermentation time (data not shown). The differences in competitiveness of rodent and sourdough isolates, however, are smaller than differences between individual strains, reflecting the relatedness of the rodent forestomach and sourdough environments^30^. Because the time between sourdough contamination with *L. reuteri* and the isolation of the specific strains is unknown, it is not possible to discriminate whether the specific differences of the sourdough isolates reflect selection for a specific subset of rodent isolates, or “reverse evolution” of a gut symbiont to food fermentations.

In conclusion, this study demonstrated that gene loss and gene gain as well as selective pressure on the core genome drive the evolution of *L. reuteri*. Remarkably, the gene content of sourdough isolates of *L. reuteri* differed from intestinal isolates, and genes under positive selection in sourdough strains included maltose phosphorylase, alcohol dehydrogenase, and lactate dehydrogenase, genes which are known to contribute to competitiveness in cereal fermentations. The study improves our understanding of the adaptation of bacteria to food fermentations as an evolutionary recent man-made habitat. It will also improve our ability to use food fermentations as model systems for more complex, intestinal ecosystems^31^.

## Methods

### Strains, media and growth conditions

The sourdough isolates *L. reuteri* LTH2584, TMW1.112, TMW1.656 and LTH5448^6^,^9^,^17^ and the rodent isolates *L. reuteri* 100-23, mlc3, and lpuph^32^ were grown anaerobically at 37 °C in mMRS^8^. Sugars were autoclaved separately. Solid media contained additional 20 g agar per liter.

### Whole-genome alignment and phylogenetics

Genome sequences of the 12 *L. reuteri* were retrieved from Genbank (Table S1). Genome sequences of sourdough isolates^16^ were re-annotated on the RAST server^33^ after gap closing by PCR amplification and Sanger sequencing. Primers binding to up- and down- stream locus of the target gap were selected after alignment of the genomes with Mauve^34^, and are shown in Table S1. Sequencing was performed by service of Macrogen Co. (Rockville, Maryland, USA).

All 16 genomes were aligned with Mugsy^35^. Homologous blocks present in each genome were concatenated with an in-house perl script. The most disordered regions were eliminated using Gblocks^36^. The disordered regions includes sites containing at least one gap, and sites that are too divergent as they may not be homologous or may be saturated by multiple substitutions. The core genome size of *L. reuteri* was about 1.2 Mbp. A maximum-likelihood core genome tree was constructed using RaxML^37^. The tree was inferred under the general time-reversible nucleotide substitution model (GTR), with gamma-distributed rate heterogeneity of four rate categories (+Γ4) (Γ4). Bootstrap support values were calculated from 1000 replicates.

### Gene clustering and construction of a gene content tree

Protein sequences longer than 50 amino acids from all genomes were combined and searched using BLAST with an all-against-all style with default parameters. The protein sequences with identities and coverage above 70% were clustered into families using the program orthoMCL^38^. The inflation value of 2 was used for the MCL clustering. Core genes were defined as those shared by all of the 16 strains; distributed genes as those shared by 2 to 15 strains, and unique genes as those only contained in one strain.

A matrix of the presence or absence of each gene for each genome was created. A dissimilarity distance between genomes based on gene content (binary data for presence or absence of each protein family) measured by one minus the Jaccard coefficient (Jaccard distance) was calculated from this matrix^39^. A gene content tree was constructed using the hierarchical clustering (UPGMA) method based on these distances by MEGA^40^.

### Analysis of positive selection

For each cluster of the single-copy core genes, protein sequences were aligned with MUSCLE^41^. These alignments were reverse-translated to codon-based nucleotide alignments by PAL2NAL^42^. Positive selection analysis based on each of these alignments was performed by CODEML implemented in PAML^43^. Nonsynonymous (amino acid altering) synonymous (silent) substitution ratios (ω), with ω = 1, < 1, or >1 indicate neutral, purifying, or positive selection, respectively. Positive selection was analysed on each family of core genes shared by all the 16 *L. reuteri* isolates using the site models M1a and M2a^44^,^45^. The model M1a (nearly neutral) allows all sites to be purifying selection (ω_0_ < 1) or neutral selection (ω_0_ = 1); the model M2a allows all sites to be positive selection (ω_0_ > 1). A likelihood ratio test (LRT) was carried out to infer the occurrence of sites subject to positive selective pressure through comparing M1a against M2a. Branch-site model and the one-ratio null model were used to analyze positive selection across the *L. reuteri* LTH2584/TWM1.112/TWM1.656 branch. Branch-site model allows ω to vary both among sites in the protein and across branches on the tree and aim to detect positive selection affecting a few sites along particular lineages (called foreground branches)^46^. Two models were used, the null model does not allow positive selection for the foreground branch, and the alternative model assumes that the foreground branch may have some sites under positive selection. For the alternative model, three classes of ω (dN/dS) were defined: ω_0_ < 1, ω_1_ = 1 and ω_2_  ≥ 1, while in the null model, ω_2_ was fixed to 1. A likelihood ratio test (LRT) was carried out to infer positive selective pressure across the *L. reuteri* LTH2584/TWM1.112/TWM1.656 branch through comparing the results from these two models. The LRT statistic (twice the log-likelihood difference between the null and the alternative models) was compared with the chi-square distribution with 2 degrees of freedom for M2a vs. M1a, and one degree of freedom for branch-site model vs. the null model.

For Clusters of Orthologous Groups of proteins (COG) analysis, we constructed a local COG database^47^, and then ran rpsblast using the sequence sets mentioned above as queries. We focused on the top three hits of each alignment and counted each category for comparison using in-house Perl script.

### Competitiveness of *L. reuteri* in sourdough: experimental design

The persistence of strains was analyzed in back-slopped rye sourdough fermentations; experiments were carried out with fermentation times of 1, 2, and 3 days. Competition experiments were carried out with six strain combinations; sourdoughs were inoculated with *L. reuteri* LTH2584 and 100-23; *L. reuteri* LTH5448 and 100-23; *L. reuteri* LTH5448 and mlc3; *L. reuteri* LTH5448 and lpuph; *L. reuteri* LTH2584 and LTH5448; or *L. reuteri* LTH2584, TMW1.112, TMW1.656, and LTH5448.

### Sourdough preparation and differential enumeration of cell counts

Competition experiments in sourdough were performed essentially as described^10^. In brief, sourdough was prepared by mixing 10 g rye flour with 10 ml of autoclaved tap water and 1 ml of bacterial inoculum. For binary and quaternary strain combinations, 0.5 and 0.25 mL, respectively, of the cell suspensions of individual strains were mixed to obtain 1 mL of bacterial cocktail as inoculum. Dough was fermented at 37 °C for 1, 2, or 3 days and back-slopped over 10 fermentation cycles. At each back-slopping step, 1 g of ripe sourdough from the previous cycle was mixed with 9.5 g of fresh rye flour and 9.5 ml of autoclaved tap water. The competition experiments were performed in duplicate and analyses were carried out with two technical replicates.

At each fermentation cycle, sourdoughs were analysed with respect to the pH, differential cell counts, and qPCR with strain specific primers. Viable cell counts were enumerated by surface-plating of appropriate dilutions on mMRS agar. Individual strains were differentiated on the basis of the colony morphology. Differential enumeration was possible for the binary strain combinations *L. reuteri* LTH2584 vs. 100-23, *L. reuteri* LTH2584 vs. LTH5448, *L. reuteri* LTH5448 vs. mlc3, and *L. reuteri* LTH5448 vs. lpuph, but not for *L. reuteri* LTH5448 vs. 100-23. In the quaternary strain combination, the combined total of *L. reuteri* LTH2584, TMW1.112 and TMW1.656 was differentiated from *L. reuteri* LTH5448.

### Analysis of sourdough microbiota by qPCR

Total DNA was isolated from sourdough^48^ and gene copy numbers of *L. reuteri* were quantified by strain-specific qPCR. Strain-specific primers are listed in Table 1. Standard curves to convert detection threshold cycles to gene copy numbers were established by analysis of 10-fold serial dilutions of target DNA of known concentration. The strain-specific primers to for strain specific quantification are shown in Table 1.

Calibration curves to convert gene copy numbers to cell counts in sourdough were Calibration curves to convert gene copy numbers to cell counts in sourdough were established with sourdoughs that were fermented with single strains. Samples were mixed with 2 volumes of sterile saline, and serially diluted with saline. From each of the dilutions, cell counts were determined by further dilution and surface plating and the gene copy numbers were quantified by qPCR as described above. Calibration curves were established in duplicate after 1 and 3 d of fermentation (Figure S2).

Quantitative PCR analyses were carried out in duplicate in MicroAmp Fast Optical 96-well reaction plates capped with MicroAmp Optical Adhesive Film (Applied Biosystems, Burlington, ON, Canada). The PCR reaction mixture consisted of 12.5 μl Fast SYBR Green Master Mix (Applied Biosystems), 0.4 μM of each primer (Table S1), 2 μl of template DNA and sterile Milli-Q water to final volume of 25 μl. Melting curves were obtained by a stepwise increase of the temperature from 60 to 95 °C at 0.05 °C/s) and melting-curve data were analyzed to verify amplification of the correct targeted PCR products. The detection limit was 10^2^ copy numbers/g sourdough for the strain-specific primers.

### Competitiveness of isogenic reutericyclin-positive and reutericyclin-negative and reutericyclin-sensitive isogenic strains of *L. reuteri*

Competition experiment between reutericyclin-positive wild type strain *L. reuteri* TMW1.656 and its reutericyclin-susceptible mutant *L. reuteri* TMW1.656*ΔrtcNΔrtcT*^16^ were performed using white wheat flour with a dough yield of 200. Sourdough was propagated every 24h with 1% inoculum for 5 days. The ratio of wild type to mutant strains at the end of each fermentation cycle was determined by qPCR with primers listed in Table 1.

### Calculation of the relative fitness of strains of *L. reuteri* in sourdough

The differential cell counts and the strain-specific gene copy numbers were used to calculate the relative fitness of the respective strains of *L. reuteri*. The fitness (w) of strain x relative to that of strain y was calculated based an equation derived from^49^:


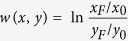


where x_0_ and y_0_ denote the strain specific cell densities or gene copy numbers at the beginning of each fermentation cycle and x_F_ and y_F_ are cell densities at the end of each of fermentation cycle. For each competition experiment, the relative fitness was plotted as average of 20 replicates (replicate experiments with 10 fermentation cycles each).

## Additional Information

**Accession numbers**: The re-annotated genomes of L. *reuteri* TMW1.112 and TMW1.656 are deposited with the Genebank accession number.

**How to cite this article**: Zheng, J. *et al.* Comparative genomics *Lactobacillus reuteri* from sourdough reveals adaptation of an intestinal symbiont to food fermentations. *Sci. Rep.* **5**, 18234; doi: 10.1038/srep18234 (2015).

## Supplementary Material

Supplementary Information

## Figures and Tables

**Table 1:** Primers used for the strain-specific PCR quantification of strains of *L. reuteri* in sourdoughs.

Target strain		Primer (5′-3)	Annealing temp. (^o^C)	Amplicon size (bp)
LTH2584^a^	84F1	GGCGTTCCTTTAACTGCTTTAAC^a^	58	95
	84R1	CTTCCTGTCCCACCAGAAATAA		
	84F2	TCTACCGGGTCTATGGCTATC^a^	58	105
	84R2	CGTTGGGCAGGGTGTAAATA		
LTH5448	48F	TAAGGCTGCTCGCAAGTATTTA	58	131
	48R	CGGTATTTGCTTTCGCACTAAC		
100-23	23F	CCTTCATCAGTCTTAGCGTCTT	60	109
	23R	GCGATAGCTGGAATGGGATTA		
TMW1.112	12F	TTGTTGTCGTTGGTGGTATGA	60	103
	12R	CCTCCAACTGCTAAACCAATCT		
TMW1.656^b^	56F	GCAGCCCAAGTAACTGAAGA	58	132
	56R	CCACCAACCAGGAAGCATAA		
	wF	GGCGGAACGTTGAATATTGT	58	248
	wR	ATTTTGGGGGAATCATAGCC		
TMW1.656Δ*rtcN*Δ*rtcT*^b^	mF	CACGTGTGTCAATAAAAAGCTGA	58	156
	mR	AACTAAACGTGCCCCATTTG		

^a^The primer pair 84F1/R1 was used for differentiation of *L. reuteri* LTH2584 from strains *L. reuteri* TMW1.112 and TMW1.656; the primer pair 84F2/R2 was used for differentiation from *L. reuteri* 100-23.

^b^The primer pairs wF/wR and mF/mR were used for quantification of *L. reuteri* TMW1.656 and *L. reuteri* TMW1.656ΔrtcNΔrtcT, respectively, in competition experiments with the wild type and mutant strains.

**Table 2:** Distributed genes specific to sourdough strains.

Gene or gene cluster	(Putative) function [ref]
**Exclusive to all sourdough strains**	
(*L. reuteri* LTH2584, TMW1.112, TMW1.656, and LTH5448)	
Reutericyclin genomic island	Reutericyclin biosynthesis and resistance [16]
Components of an ABC transporter	Unknown [16]
Aspartate racemase (WP_003670574.1),	
LytTr DNA-binding domain (WP_006729038.1)	Unknown
**Exclusive to some sourdough strains:**	
*L. reuteri* LTH2584 and TMW1.112	
GntR (WP_006916030.1),	
Membrane transport protein (WP_006916028.1);	Unknown
Hydroxyglutarate dehydrogenase (WP_006916027.1)	Use of ketoglutarate as electron acceptor [19]
*L. reuteri* LTH2584 and TMW1.656	
Two GT8_A4GalT_like proteins (WP_020807748.1, WP_020807748.1),	Bread spectrum glycosyltransferases with putative function in protein glycosylation;
YkuD (WP_003664366.1)	peptidoglycan crosslinking
**Predominantly sourdough strains**	
*L. reuteri* TMW1.112, TMW1.656 and mlc3	
mlc3|WP_019251925.1, WP_019251926.1, WP_019251927.1, WP_019251928.1; WP_019251930.1; WP_019251931.1; WP_019251932.1; WP_019251933.1	Putative components of shikimic acid pathway for biosynthesis of aromatic amino acids
*L. reuteri* TMW1.112, LTH5448 and 100-23	
Homocysteine methyltransferase (100_23|ZP_03072304)	
S-methylmethionine transporter (100_23|ZP_03072305)	Oxidative stress response
*L. reuteri* LTH2584, TMW1.112, TMW1.656, and 100-23	
Lr100-23|ZP_03073418.1|	NADPH-dependent FMN reductase. rRNA
Lr100-23|ZP_03073416.1|	methyltransferase
Lr100-23|ZP_03073414.1|	Lipid metabolism]\
Lr100-23|ZP_03073413.1|	serine protease
Lr100-23|ZP_03073412.1|	Metal-dependent beta-lactamase superfamily I
Lr100-23|ZP_03073411.1|	unknown
Lr100-23|ZP_03073410.1|	unknown
Lr100-23|ZP_03073409.1|	Histidine kinase
Lr100-23|ZP_03073446.1|	Cardiolipin synthase
Lr100-23|ZP_03073445.1|	Acetyltransferase (GNAT) family

Hypothetical proteins and phage-related proteins were excluded from the list. Protein Accession numbers refer to the genome of *L. reuteri* LTH2584 unless otherwise specified.
