# Supplementary material for: Comparative genomics Lactobacillus reuteri from sourdough reveals adaptation of an intestinal symbiont to food fermentations
Source: Sci Rep. 2015 Dec 11;5:18234. doi: 10.1038/srep18234 (PMC4995734; doi:10.1038/srep18234)
Supplement: Supplementary Information [file srep18234-s1.doc]

**Comparative genomics *Lactobacillus reuteri* from sourdough reveals adaptation of an intestinal symbiont to food fermentations**

**Jinshui Zheng, Xin Zhao, Xiaoxi B. Lin, Michael Gänzle**

**Supplementary Data**

**Table S1.** Primer sequences used for closing of gaps in the whole genome shotgun sequences

**Table S2.** Genome characteristics of strains used in this study.

**Table S3** Genes under positive selection of the 16 strains

**Table S4** Genes under positive selection of the 4 sourdough strains

**Figure S1.** Visual representation of the core genome and the pan genome of *L. reuteri.*

**Figure S2.** Calibration curves to convert qPCR data (gene copy numbers) to cell counts

**Figure S3.** Cell density of individual *L. reuteri* isolates in competition experiments in sourdough.

**Figure S4.** pH value of sourdoughs fermented with binary or quarternary strain combinations

**Figure S5.** Total viable plate counts of sourdoughs fermented with binary or quaternary strain combinations.

**Table S1.** Primers for closing of gaps in the whole genome shotgun sequences of *L. reuteri* TMW1.112 and *L. reuteri* TMW1.656.

| **Gap within scaffold** | **Primers (5'-3')(forward / reverse)** | **Elongation time (sec)** |
| --- | --- | --- |
| *L. reuteri* TMW1.112 | | |
| Gap A1+A2 | For: CACACCAAATTCGCGTTC | 220 |
|  | Rev: TGCTTAATGCGTTAGCTC |  |
| Gap A3 | For: TACCCGAAAGTAGTCGAG | 150 |
|  | Rev: CTTCCGCAAAATAGGTGT |  |
| Gap A4 | For: CTTCCGCAAAATAGGTGT | 150 |
|  | Rev: GATGGTGCAGTCTTGGG |  |
| Gap A5 | For: CTTTAGCACTCCGCATT | 90 |
|  | Rev: AAGAACGTGGTAATGCTC |  |
| Gap A6 | For: ATTCGGGTCCTCAACCAC | 90 |
|  | Rev: GCTGACCGCTTGTCGAG |  |
| Gap A14 | For: AGGTTATGCTTACGGTCA | 90 |
|  | Rev: ACGGAAGTTCGACGTTG |  |
| Gap A15+A16+A17 | For: ATTTTCCACAGCCCGTTG | 220 |
|  | Rev: TCCAAAATTAACCGCTCT |  |
| Gap A18+19 | For: CTGCGAATTAAATTGGTC | 120 |
|  | Rev: CGCAAATATCTGTACCCT |  |
| *L. reuteri* TMW1.656 | | |
| Gap A1 | For: TAGTCGCCAATAATCGAG | 260 |
|  | Rev: TGCATCATTTAAGCACCT |  |
| Gap A3 | For: AACCAAATCAGCGAGCAG | 120 |
|  | Rev: CAGCCCATTGAAGGGTT |  |
| Gap A4 | For: TGAAACGGTAATGCAAG | 220 |
|  | Rev: GCACGATCATAATTGGG |  |
| Gap A7 | For: CATAATGATATAGCCCTGT | 180 |
|  | Rev: CAACGTGGACTAGAACCC |  |
| Gap A8 | For: TGGAAATAGTTGCCCGTA | 160 |
|  | Rev: TAATTATGCGGTGACTGG |  |

**Table S2. Genome characteristics of strains used in this study.**

| **Strain number** | **Accession number** | **# of contigs** |
| --- | --- | --- |
| DSM 20016 | [NC_009513.1](http://www.ncbi.nlm.nih.gov/nuccore/148543243) | 1 |
| JCM 1112 | [NC_010609.1](http://www.ncbi.nlm.nih.gov/nuccore/184152655) | 1 |
| SD2112 | [NC_015697.1](http://www.ncbi.nlm.nih.gov/nuccore/338202359); [NC_015700.1](http://www.ncbi.nlm.nih.gov/nuccore/338202319); [NC_015698.1](http://www.ncbi.nlm.nih.gov/nuccore/338202336); [NC_015701.1](http://www.ncbi.nlm.nih.gov/nuccore/338204606); [NC_015699.1](http://www.ncbi.nlm.nih.gov/nuccore/338202351) | 1 chromosome, 4 plasmids |
| I5007 | [NC_021494.1](http://www.ncbi.nlm.nih.gov/nuccore/512590512); [NC_021503.1](http://www.ncbi.nlm.nih.gov/nuccore/512656346); [NC_021495.1](http://www.ncbi.nlm.nih.gov/nuccore/512593096); [NC_021496.1](http://www.ncbi.nlm.nih.gov/nuccore/512594165); [NC_021497.1](http://www.ncbi.nlm.nih.gov/nuccore/512595024); [NC_021504.1](http://www.ncbi.nlm.nih.gov/nuccore/512657636); [NC_021498.1](http://www.ncbi.nlm.nih.gov/nuccore/512595901) | 1 chromosome, 6 plasmids |
| TD1 | [NC_021872.1](http://www.ncbi.nlm.nih.gov/nuccore/526230725) | 1 |
| CF48-3A | [NZ_ACHG00000000.1](http://www.ncbi.nlm.nih.gov/nuccore/224922074) | 92 |
| MM2-3 | [NZ_ACLB00000000.1](http://www.ncbi.nlm.nih.gov/nuccore/227365236) | 95 |
| ATCC 53608 | [NZ_CACS00000000.2](http://www.ncbi.nlm.nih.gov/nuccore/423332544) | 64 |
| MM4-1A | |  | [NZ_ACGX00000000.2](http://www.ncbi.nlm.nih.gov/nuccore/325683662) | | --- | --- | | 7 |
| 100-23 | [NZ_AAPZ00000000.2](http://www.ncbi.nlm.nih.gov/nuccore/194468466) | 2 |
| mlc3 | |  | [NZ_AEAW00000000.1](http://www.ncbi.nlm.nih.gov/nuccore/485035114) | | --- | --- | | 126 |
| Lpuph | [NZ_AEAX00000000.1](http://www.ncbi.nlm.nih.gov/nuccore/485035242) | 127 |
| LTH2484 | [NZ_JOSX00000000.1](http://www.ncbi.nlm.nih.gov/nuccore/737184073) | 25 |
| TMW1.656 | NZ_JOSW00000000.1 | 17 |
| TMW1.112 | [NZ_JOKX00000000.1](http://www.ncbi.nlm.nih.gov/nuccore/737168061) | 12 |
| LTH5448 | NZ_JOOG00000000.1 | 36 |

**Table S2**. Genes under positive selection in the 16 genomes of *L. reuteri*

| **Protein Acc. No  in 100-23** | **COG** | **COG category** | **Function** |
| --- | --- | --- | --- |
| WP_003666558.1 | COG0356 | C | F0F1-type ATP synthase, subunit a |
| WP_003665654.1 | COG0037 | D | tRNA(Ile)-lysidine synthase MesJ |
| WP_003666765.1 | COG0849 | D | Cell division ATPase FtsA |
| WP_003665400.1 | COG1296 | E | Predicted branched-chain amino acid permease (azaleucine resistance) |
| WP_003666360.1 | COG0263 | E | Glutamate 5-kinase |
| WP_003666409.1 | COG0624 | E | Acetylornithine deacetylase/Succinyl-diaminopimelate desuccinylase and related deacylases |
| WP_003666512.1 | COG0078 | E | Ornithine carbamoyltransferase |
| WP_003666614.1 | COG0028 | EH | Thiamine pyrophosphate-requiring enzymes |
| WP_003666411.1 | COG0834 | ET | ABC-type amino acid transport/signal transduction systems, periplasmic component/domain |
| WP_003665376.1 | COG1957 | F | Inosine-uridine nucleoside N-ribohydrolase |
| WP_003665434.1 | COG0046 | F | Phosphoribosylformylglycinamidine (FGAM) synthase, synthetase domain |
| WP_003666321.1 | COG0209 | F | Ribonucleotide reductase, alpha subunit |
| WP_003665438.1 | COG0150 | F | Phosphoribosylaminoimidazole (AIR) synthetase |
| WP_003666060.1 | COG0207 | F | Thymidylate synthase |
| WP_003665127.1 | COG0518 | F | GMP synthase - Glutamine amidotransferase domain |
| WP_003665407.1 | COG0044 | F | Dihydroorotase and related cyclic amidohydrolases |
| WP_003666124.1 | COG1957 | F | Inosine-uridine nucleoside N-ribohydrolase |
| WP_003664237.1 | COG2814 | G | Arabinose efflux permease |
| WP_003666072.1 | COG2017 | G | Galactose mutarotase and related enzymes |
| WP_003665682.1 | COG0061 | G | NAD kinase |
| WP_003663649.1 | COG0697 | GER | Permeases of the drug/metabolite transporter (DMT) superfamily |
| WP_003663861.1 | COG0142 | H | Geranylgeranyl pyrophosphate synthase |
| WP_003664178.1 | COG0294 | H | Dihydropteroate synthase and related enzymes |
| WP_003664181.1 | COG0285 | H | Folylpolyglutamate synthase |
| WP_003664112.1 | COG0237 | H | Dephospho-CoA kinase |
| WP_003664185.1 | COG0801 | H | 7,8-dihydro-6-hydroxymethylpterin-pyrophosphokinase |
| WP_003664677.1 | COG0657 | I | Esterase/lipase |
| WP_003663678.1 | COG0825 | I | Acetyl-CoA carboxylase alpha subunit |
| WP_003665328.1 | COG0183 | I | Acetyl-CoA acetyltransferase |
| WP_003663677.1 | COG0777 | I | Acetyl-CoA carboxylase beta subunit |
| WP_003664573.1 | COG0480 | J | Translation elongation factors (GTPases) |
| WP_003665683.1 | COG0564 | J | Pseudouridylate synthases, 23S RNA-specific |
| WP_003664908.1 | COG0480 | J | Translation elongation factors (GTPases) |
| WP_003664503.1 | COG0721 | J | Asp-tRNAAsn/Glu-tRNAGln amidotransferase C subunit |
| WP_003666543.1 | COG2890 | J | Methylase of polypeptide chain release factors |
| WP_003664494.1 | COG2265 | J | SAM-dependent methyltransferases related to tRNA (uracil-5-)-methyltransferase |
| WP_003664519.1 | COG0101 | J | Pseudouridylate synthase |
| WP_003665664.1 | COG1190 | J | Lysyl-tRNA synthetase (class II) |
| WP_003665361.1 | COG2265 | J | SAM-dependent methyltransferases related to tRNA (uracil-5-)-methyltransferase |
| WP_003666200.1 | COG0564 | J | Pseudouridylate synthases, 23S RNA-specific |
| WP_003666676.1 | COG0343 | J | Queuine/archaeosine tRNA-ribosyltransferase |
| WP_003665800.1 | COG0858 | J | Ribosome-binding factor A |
| WP_003665090.1 | COG0441 | J | Threonyl-tRNA synthetase |
| WP_003666455.1 | COG1670 | J | Acetyltransferases, including N-acetylases of ribosomal proteins |
| WP_003664575.1 | COG0049 | J | Ribosomal protein S7 |
| WP_003664569.1 | COG0051 | J | Ribosomal protein S10 |
| WP_003664006.1 | COG0211 | J | Ribosomal protein L27 |
| WP_003666161.1 | COG0144 | J | tRNA and rRNA cytosine-C5-methylases |
| WP_003664517.1 | COG0102 | J | Ribosomal protein L13 |
| WP_003665822.1 | COG1490 | J | D-Tyr-tRNAtyr deacylase |
| WP_003664993.1 | COG1476 | K | Predicted transcriptional regulators |
| WP_003664629.1 | COG0789 | K | Predicted transcriptional regulators |
| WP_003666175.1 | COG1609 | K | Transcriptional regulators |
| WP_003665403.1 | COG0583 | K | Transcriptional regulator |
| WP_003666533.1 | COG1438 | K | Arginine repressor |
| WP_003666847.1 | COG0608 | L | Single-stranded DNA-specific exonuclease |
| WP_003663796.1 | COG0178 | L | Excinuclease ATPase subunit |
| WP_003665002.1 | COG2094 | L | 3-methyladenine DNA glycosylase |
| WP_003663571.1 | COG0419 | L | ATPase involved in DNA repair |
| WP_003666195.1 | COG0116 | L | Predicted N6-adenine-specific DNA methylase |
| WP_003664214.1 | COG2818 | L | 3-methyladenine DNA glycosylase |
| WP_003663858.1 | COG0497 | L | ATPase involved in DNA repair |
| WP_003664638.1 | COG1573 | L | Uracil-DNA glycosylase |
| WP_003666672.1 | COG0323 | L | DNA mismatch repair enzyme (predicted ATPase) |
| WP_003666649.1 | COG2003 | L | DNA repair proteins |
| WP_003665027.1 | COG0791 | M | Cell wall-associated hydrolases (invasion-associated proteins) |
| WP_003666800.1 | COG0791 | M | Cell wall-associated hydrolases (invasion-associated proteins) |
| WP_003664439.1 | COG5632 | M | N-acetylmuramoyl-L-alanine amidase |
| WP_003665598.1 | COG0766 | M | UDP-N-acetylglucosamine enolpyruvyl transferase |
| WP_003665614.1 | COG0770 | M | UDP-N-acetylmuramyl pentapeptide synthase |
| WP_003666198.1 | COG0597 | MU | Lipoprotein signal peptidase |
| WP_003664205.1 | COG1705 | NU | Muramidase (flagellum-specific) |
| WP_003666352.1 | COG1214 | O | Inactive homolog of metal-dependent proteases, putative molecular chaperone |
| WP_003665807.1 | COG0576 | O | Molecular chaperone GrpE (heat shock protein) |
| WP_003664641.1 | COG0471 | P | Di- and tricarboxylate transporters |
| WP_003664764.1 | COG1464 | P | ABC-type metal ion transport system, periplasmic component/surface antigen |
| WP_003664880.1 | COG1122 | P | ABC-type cobalt transport system, ATPase component |
| WP_003664817.1 | COG2262 | R | GTPases |
| WP_003666191.1 | COG3331 | R | Penicillin-binding protein-related factor A, putative recombinase |
| WP_003664848.1 | COG2936 | R | Predicted acyl esterases |
| WP_003666665.1 | COG1058 | R | Predicted nucleotide-utilizing enzyme related to molybdopterin-biosynthesis enzyme MoeA |
| WP_003666828.1 | COG2333 | R | Predicted hydrolase (metallo-beta-lactamase superfamily) |
| WP_003663828.1 | COG1461 | R | Predicted kinase related to dihydroxyacetone kinase |
| WP_003666365.1 | COG0488 | R | ATPase components of ABC transporters |
| WP_003664976.1 | COG0656 | R | Aldo/keto reductases, related to diketogulonate reductase |
| WP_003666471.1 | COG0561 | R | Predicted hydrolases of the HAD superfamily |
| WP_003666484.1 | COG0110 | R | Acetyltransferase (isoleucine patch superfamily) |
| WP_003666380.1 | COG1040 | R | Predicted amidophosphoribosyltransferases |
| WP_003664926.1 | COG0628 | R | Predicted permease, member of the PurR regulon |
| WP_003665008.1 | COG2249 | R | Putative NADPH-quinone reductase (modulator of drug activity B) |
| WP_003666217.1 | COG0456 | R | Acetyltransferases |
| WP_003666258.1 | COG2985 | R | Predicted permease |
| WP_003666215.1 | COG4814 | R | Uncharacterized protein with an alpha/beta hydrolase fold |
| WP_003663772.1 | COG0561 | R | Predicted hydrolases of the HAD superfamily |
| WP_003663776.1 | COG4814 | R | Uncharacterized protein with an alpha/beta hydrolase fold |
| WP_003665364.1 | COG0431 | R | Predicted flavoprotein |
| WP_003664885.1 | COG1073 | R | Hydrolases of the alpha/beta superfamily |
| WP_003664349.1 | COG4684 | S | Predicted membrane protein |
| WP_003664422.1 | COG2246 | S | Predicted membrane protein |
| WP_003665129.1 | COG4841 | S | Uncharacterized protein conserved in bacteria |
| WP_003666456.1 | COG2461 | S | Uncharacterized conserved protein |
| WP_003664210.1 | COG1598 | S | Predicted nuclease of the RNAse H fold, HicB family |
| WP_003665003.1 | COG3189 | S | Uncharacterized conserved protein |
| WP_003666105.1 | COG3152 | S | Predicted membrane protein |
| WP_003664609.1 | COG1814 | S | Uncharacterized membrane protein |
| WP_003666679.1 | COG1862 | U | Preprotein translocase subunit YajC |
| WP_003663819.1 | COG0552 | U | Signal recognition particle GTPase |
| WP_003665010.1 | - | - | hypothetical protein |
| WP_003664831.1 | - | - | hypothetical protein |
| WP_003665115.1 | - | - | hypothetical protein |
| WP_003664438.1 | - | - | hypothetical protein |
| WP_003664432.1 | - | - | hypothetical protein |
| WP_003664768.1 | - | - | hypothetical protein |
| WP_003664021.1 | - | - | hypothetical protein |
| WP_003666316.1 | - | - | hypothetical protein |
| WP_003664935.1 | - | - | hypothetical protein |
| WP_003666693.1 | - | - | hypothetical protein |
| WP_003665064.1 | - | - | hypothetical protein |
| WP_003664971.1 | - | - | hypothetical protein |
| WP_003666804.1 | - | - | hypothetical protein |
| WP_003665000.1 | - | - | hypothetical protein |
| WP_003664232.1 | - | - | hypothetical protein |
| WP_003666047.1 | - | - | hypothetical protein |
| WP_003664174.1 | - | - | hypothetical protein |

**Table S3**. Genes under positive selection of the 3 lineage II sourdough isolates

| **Protein Acc. Number in 100-23** | | **COG** | | **COG category** | | **Function** | |
| --- | --- | --- | --- | --- | --- | --- | --- |
| WP_003665061.1 | COG0371 | | C | | Glycerol dehydrogenase and related enzymes | |  |
| WP_003666182.1 | COG1304 | | C | | Isopentenyl diphosphate isomerase (BS_ypgA, MTH48 and related proteins) | |  |
| WP_003666317.1 | COG1454 | | C | | Alcohol dehydrogenase, class IV | |  |
| WP_003666809.1 | COG0022 | | C | | Pyruvate/2-oxoglutarate dehydrogenase complex, dehydrogenase (E1) component, eukaryotic type, beta subunit | |  |
| WP_003663509.1 | COG0554 | | C | | Glycerol kinase | |  |
| WP_003665460.1 | COG1757 | | C | | Na+/H+ antiporter | |  |
| WP_003664644.1 | COG0281 | | C | | Malic enzyme | |  |
| WP_003664822.1 | COG1052 | | CHR | | Lactate dehydrogenase | |  |
| WP_003666646.1 | COG4987 | | CO | | ABC-type transport system involved in cytochrome bd biosynthesis, fused ATPase and permease components | |  |
| WP_003666634.1 | COG4477 | | D | | Negative regulator of septation ring formation | |  |
| WP_003665488.1 | COG1174 | | E | | ABC-type proline/glycine betaine transport systems, permease component | |  |
| WP_003665217.1 | COG0367 | | E | | Asparagine synthase (glutamine-hydrolyzing) | |  |
| WP_003666793.1 | COG0289 | | E | | Dihydrodipicolinate reductase | |  |
| WP_003666790.1 | COG2171 | | E | | Tetrahydrodipicolinate N-succinyltransferase | |  |
| WP_003666789.1 | COG0019 | | E | | Diaminopimelate decarboxylase | |  |
| WP_003666795.1 | COG0136 | | E | | Aspartate-semialdehyde dehydrogenase | |  |
| WP_003663503.1 | COG4690 | | E | | Dipeptidase | |  |
| WP_003666788.1 | COG0527 | | E | | Aspartokinases | |  |
| WP_003663799.1 | COG0346 | | E | | Lactoylglutathione lyase and related lyases | |  |
| WP_003666203.1 | COG0458 | | EF | | Carbamoylphosphate synthase large subunit (split gene in MJ) | |  |
| WP_003666792.1 | COG0329 | | EM | | Dihydrodipicolinate synthase/N-acetylneuraminate lyase | |  |
| WP_003665343.1 | COG0516 | | F | | IMP dehydrogenase/GMP reductase | |  |
| WP_003664513.1 | COG0503 | | F | | Adenine/guanine phosphoribosyltransferases and related PRPP-binding proteins | |  |
| WP_003665442.1 | COG0138 | | F | | AICAR transformylase/IMP cyclohydrolase PurH (only IMP cyclohydrolase domain in Aful) | |  |
| WP_003665425.1 | COG0026 | | F | | Phosphoribosylaminoimidazole carboxylase (NCAIR synthetase) | |  |
| WP_003664874.1 | COG1957 | | F | | Inosine-uridine nucleoside N-ribohydrolase | |  |
| WP_003666778.1 | COG0775 | | F | | Nucleoside phosphorylase | |  |
| WP_003664512.1 | COG0026 | | F | | Phosphoribosylaminoimidazole carboxylase (NCAIR synthetase) | |  |
| WP_003664236.1 | COG1328 | | F | | Oxygen-sensitive ribonucleoside-triphosphate reductase | |  |
| WP_003666048.1 | COG0283 | | F | | Cytidylate kinase | |  |
| WP_003666825.1 | COG2131 | | F | | Deoxycytidylate deaminase | |  |
| WP_003666786.1 | COG0462 | | FE | | Phosphoribosylpyrophosphate synthetase | |  |
| WP_003666231.1 | COG2814 | | G | | Arabinose efflux permease | |  |
| WP_003665600.1 | COG1455 | | G | | Phosphotransferase system cellobiose-specific component IIC | |  |
| WP_003665332.1 | COG1554 | | G | | Maltose phosphorylase | |  |
| WP_003666179.1 | COG3345 | | G | | Alpha-galactosidase | |  |
| WP_003666129.1 | COG2271 | | G | | Sugar phosphate permease | |  |
| WP_003666465.1 | COG1109 | | G | | Phosphomannomutase | |  |
| WP_003666099.1 | COG2610 | | GE | | H+/gluconate symporter and related permeases | |  |
| WP_003663565.1 | COG4464 | | GM | | Capsular polysaccharide biosynthesis protein | |  |
| WP_003664212.1 | COG0192 | | H | | S-adenosylmethionine synthetase | |  |
| WP_003664171.1 | COG0351 | | H | | Hydroxymethylpyrimidine/phosphomethylpyrimidine kinase | |  |
| WP_003666061.1 | COG0262 | | H | | Dihydrofolate reductase | |  |
| WP_003663791.1 | COG1154 | | HI | | Deoxyxylulose-5-phosphate synthase | |  |
| WP_003666183.1 | COG1577 | | I | | Mevalonate kinase | |  |
| WP_003666184.1 | COG3407 | | I | | Mevalonate pyrophosphate decarboxylase | |  |
| WP_003664987.1 | COG1502 | | I | | Phosphatidylserine/phosphatidylglycerophosphate/cardiolipin synthases and related enzymes | |  |
| WP_003666185.1 | COG1577 | | I | | Mevalonate kinase | |  |
| WP_003664876.1 | COG1028 | | IQR | | Dehydrogenases with different specificities (related to short-chain alcohol dehydrogenases) | |  |
| WP_003666802.1 | COG1597 | | IR | | Sphingosine kinase and enzymes related to eukaryotic diacylglycerol kinase | |  |
| WP_003664099.1 | COG1534 | | J | | Predicted RNA-binding protein containing KH domain, possibly ribosomal | |  |
| WP_003664211.1 | COG0495 | | J | | Leucyl-tRNA synthetase | |  |
| WP_003664555.1 | COG0197 | | J | | Ribosomal protein L16/L10E | |  |
| WP_003666774.1 | COG0060 | | J | | Isoleucyl-tRNA synthetase | |  |
| WP_003665453.1 | COG0162 | | J | | Tyrosyl-tRNA synthetase | |  |
| WP_003664420.1 | COG0024 | | J | | Methionine aminopeptidase | |  |
| WP_003663844.1 | COG0144 | | J | | tRNA and rRNA cytosine-C5-methylases | |  |
| WP_003666836.1 | COG0050 | | J | | GTPases - translation elongation factors | |  |
| WP_003665806.1 | COG1420 | | K | | Transcriptional regulator of heat shock gene | |  |
| WP_003665864.1 | COG0568 | | K | | DNA-directed RNA polymerase, sigma subunit (sigma70/sigma32) | |  |
| WP_003665360.1 | COG1609 | | K | | Transcriptional regulators | |  |
| WP_003663822.1 | COG0571 | | K | | dsRNA-specific ribonuclease | |  |
| WP_003665379.1 | COG1475 | | K | | Stage 0 sporulation protein J (antagonist of Soj) containing ParB-like nuclease domain | |  |
| WP_003663849.1 | COG1198 | | L | | Primosomal protein N' (replication factor Y) - superfamily II helicase | |  |
| WP_003666046.1 | COG0514 | | L | | Superfamily II DNA helicase | |  |
| WP_003666068.1 | COG0164 | | L | | Ribonuclease HII | |  |
| WP_003666675.1 | COG2255 | | L | | Holliday junction resolvasome, helicase subunit | |  |
| WP_003665794.1 | COG2176 | | L | | DNA polymerase III, alpha subunit (gram-positive type) | |  |
| WP_003664114.1 | COG0749 | | L | | DNA polymerase I - 3'-5' exonuclease and polymerase domains | |  |
| WP_003664109.1 | COG3611 | | L | | Replication initiation/membrane attachment protein | |  |
| WP_003664108.1 | COG1484 | | L | | DNA replication protein | |  |
| WP_003666331.1 | COG0353 | | L | | Recombinational DNA repair protein (RecF pathway) | |  |
| WP_003666843.1 | COG0322 | | L | | Nuclease subunit of the excinuclease complex | |  |
| WP_003666785.1 | COG0507 | | L | | ATP-dependent exoDNAse (exonuclease V), alpha subunit - helicase superfamily I member | |  |
| WP_003665646.1 | COG1197 | | LK | | Transcription-repair coupling factor (superfamily II helicase) | |  |
| WP_003664424.1 | COG0513 | | LKJ | | Superfamily II DNA and RNA helicases | |  |
| WP_003666070.1 | COG0758 | | LU | | Predicted Rossmann fold nucleotide-binding protein involved in DNA uptake | |  |
| WP_003666313.1 | COG1970 | | M | | Large-conductance mechanosensitive channel | |  |
| WP_003664075.1 | COG0791 | | M | | Cell wall-associated hydrolases (invasion-associated proteins) | |  |
| WP_003665812.1 | COG0481 | | M | | Membrane GTPase LepA | |  |
| WP_003664166.1 | COG0744 | | M | | Membrane carboxypeptidase (penicillin-binding protein) | |  |
| WP_003666285.1 | COG1066 | | O | | Predicted ATP-dependent serine protease | |  |
| WP_003665710.1 | COG0450 | | O | | Peroxiredoxin | |  |
| WP_003665585.1 | COG1108 | | P | | ABC-type Mn2+/Zn2+ transport systems, permease components | |  |
| WP_003665584.1 | COG1121 | | P | | ABC-type Mn/Zn transport systems, ATPase component | |  |
| WP_003665505.1 | COG1135 | | P | | ABC-type metal ion transport system, ATPase component | |  |
| WP_003664851.1 | COG0783 | | P | | DNA-binding ferritin-like protein (oxidative damage protectant) | |  |
| WP_003664521.1 | COG0619 | | P | | ABC-type cobalt transport system, permease component CbiQ and related transporters | |  |
| WP_003665028.1 | COG2984 | | R | | ABC-type uncharacterized transport system, periplasmic component | |  |
| WP_003664082.1 | COG1418 | | R | | Predicted HD superfamily hydrolase | |  |
| WP_003664101.1 | COG1161 | | R | | Predicted GTPases | |  |
| WP_003664648.1 | COG1064 | | R | | Zn-dependent alcohol dehydrogenases | |  |
| WP_003666059.1 | COG0488 | | R | | ATPase components of ABC transporters with duplicated ATPase domains | |  |
| WP_003666235.1 | COG0661 | | R | | Predicted unusual protein kinase | |  |
| WP_003664911.1 | COG0596 | | R | | Predicted hydrolases or acyltransferases (alpha/beta hydrolase superfamily) | |  |
| WP_003665414.1 | COG0637 | | R | | Predicted phosphatase/phosphohexomutase | |  |
| WP_003665032.1 | COG4120 | | R | | ABC-type uncharacterized transport system, permease component | |  |
| WP_003665591.1 | COG1078 | | R | | HD superfamily phosphohydrolases | |  |
| WP_003665468.1 | COG4989 | | R | | Predicted oxidoreductase | |  |
| WP_003666680.1 | COG0618 | | R | | Exopolyphosphatase-related proteins | |  |
| WP_003665130.1 | COG0656 | | R | | Aldo/keto reductases, related to diketogulonate reductase | |  |
| WP_003664635.1 | COG0693 | | R | | Putative intracellular protease/amidase | |  |
| WP_003665035.1 | COG1101 | | R | | ABC-type uncharacterized transport system, ATPase component | |  |
| WP_003663793.1 | COG3410 | | S | | Uncharacterized conserved protein [Function unknown] | |  |
| WP_003664639.1 | COG2966 | | S | | Uncharacterized conserved protein | |  |
| WP_003666772.1 | COG2302 | | S | | Uncharacterized conserved protein, contains S4-like domain | |  |
| WP_003666127.1 | COG4485 | | S | | Predicted membrane protein | |  |
| WP_003664133.1 | COG5584 | | S | | Predicted small secreted protein | |  |
| WP_003664998.1 | COG3864 | | S | | Uncharacterized protein conserved in bacteria | |  |
| WP_003666192.1 | COG4474 | | S | | Uncharacterized protein conserved in bacteria | |  |
| WP_003664634.1 | COG2898 | | S | | Uncharacterized conserved protein | |  |
| WP_003664653.1 | COG4320 | | S | | Uncharacterized protein conserved in bacteria | |  |
| WP_003663831.1 | COG1302 | | S | | Uncharacterized protein conserved in bacteria | |  |
| WP_003664958.1 | COG0586 | | S | | Uncharacterized membrane-associated protein | |  |
| WP_003665876.1 | COG2996 | | S | | Predicted RNA-bindining protein (contains S1 and HTH domains) | |  |
| WP_003665610.1 | COG0394 | | T | | Protein-tyrosine-phosphatase | |  |
| WP_003664953.1 | COG0589 | | T | | Universal stress protein UspA and related nucleotide-binding proteins | |  |
| WP_003666821.1 | COG3480 | | T | | Predicted secreted protein containing a PDZ domain | |  |
| WP_003666394.1 | COG1493 | | T | | Serine kinase of the HPr protein, regulates carbohydrate metabolism | |  |
| WP_003665820.1 | COG0317 | | TK | | Guanosine polyphosphate pyrophosphohydrolases/synthetases | |  |
| WP_003666531.1 | COG1680 | | V | | Beta-lactamase class C and other penicillin binding proteins | |  |
| WP_003665254.1 | COG1132 | | V | | ABC-type multidrug transport system, ATPase and permease components | |  |
| WP_003665082.1 | COG1136 | | V | | ABC-type antimicrobial peptide transport system, ATPase component | |  |
| WP_003664219.1 | COG4767 | | V | | Glycopeptide antibiotics resistance protein | |  |
| WP_003665469.1 | - | | - | | hypothetical protein | |  |
| WP_003666270.1 | - | | - | | hypothetical protein | |  |
| WP_003664842.1 | - | | - | | hypothetical protein | |  |
| WP_003665076.1 | - | | - | | hypothetical protein | |  |
| WP_003666708.1 | - | | - | | hypothetical protein | |  |
| WP_003666333.1 | - | | - | | hypothetical protein | |  |
| WP_003665706.1 | - | | - | | hypothetical protein | |  |
| WP_003664651.1 | - | | - | | hypothetical protein | |  |
| WP_003665589.1 | - | | - | | hypothetical protein | |  |

**Figure S1.** Visual representation of the core genome and the pan genome of *L. reuteri.* The figure was generated with the PanGP software (<http://sourceforge.net/projects/pangp/>)


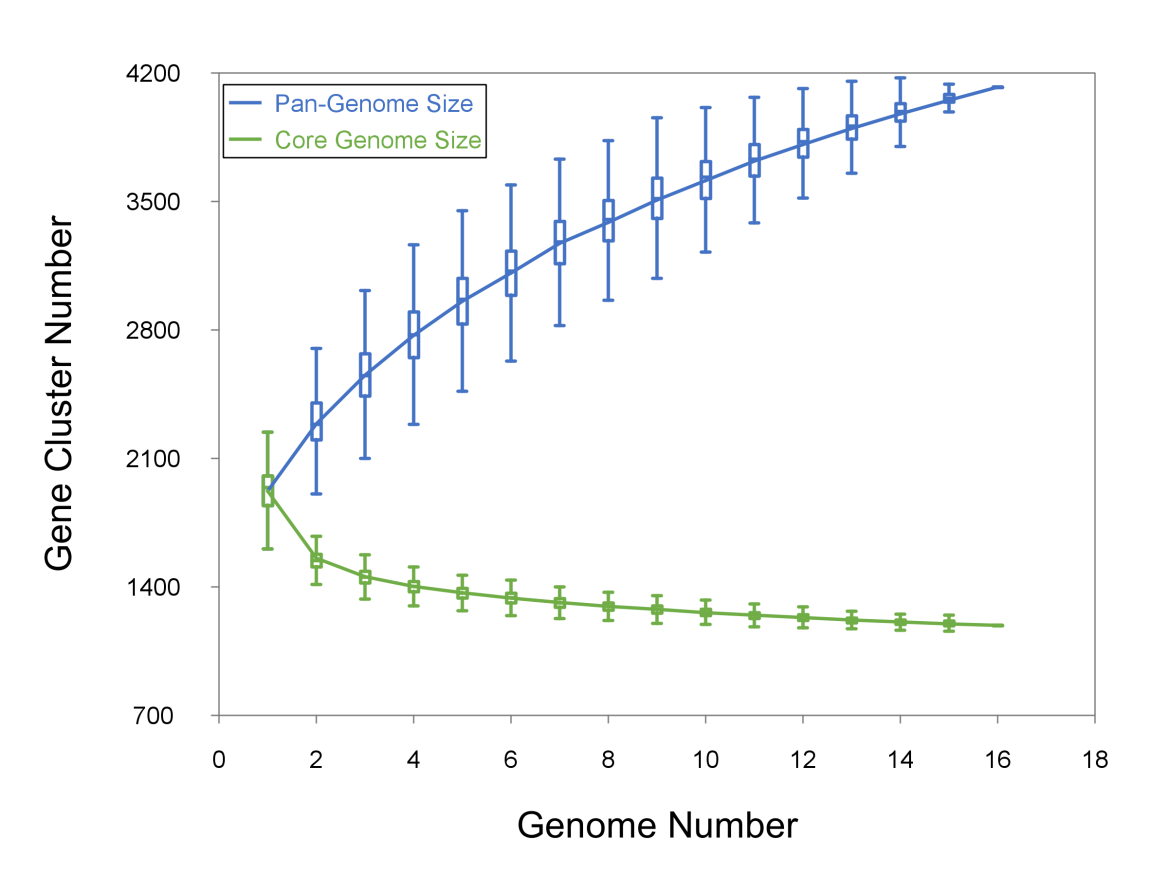


**Figure S2.** Calibration curves to convert qPCR data (gene copy numbers) to cell counts in rye sourdough. Calibration curves were established by analysis of sourdoughs after 24 h of fermentation (**black symbols)** or after 72 h of fermentation (**white symbols). Panel A:** *L. reuteri* LTH2584, LTH5448; TMW1.112 and TMW 1.656. **Panel B:** *L. reuteri* 100-23. Note the x-axis of Panel B is scaled differently, reflecting that recovery of DNA from strain 100-23 was very poor when compared to other strains. Data are shown as means ± standard deviation of triplicate or quadruplicate independent experiments.

**Figure S3. Cell density of individual *L. reuteri* isolates in competition experiments in sourdough.**

Evolution of fermentation microbiota in binary or quarternary strain competitions of strains of *L. reuteri*. Sourdoughs were inoculated with specific strains maintained by continuous back-slopping with 10% inoculum over 10 fermentation cycles with 1 d, 2 d, or 3 d incubation times. Sourdough microbiota were analysed by differential plate counts and results were expressed as log proportion of the individual strains to the total viable cell counts. Sourdoughs were also analysed by qPCR targeting strain-specific sequences and log DNA copy numbers converted to cell counts using the strain-specific calibration curves and results were expressed as proportion of the individual strains to the total cell counts.

Panels are labeled with numbers and letters. The numbers indicate the fermentation time in days, capital letters indicate panels showing differential plate counts, and lowercase letters indicate panels showing results of strain specific qPCR analysis. Letters indicate the strains used in the competition as follows: A, a: *L. reuteri* LTH2584 (■) versus 100-23 (○); B, b: LTH5448 (▲) versus 100-23 (○); C, c: *L. reuteri* LTH2584 (■) versus LTH5448 (▲); D, d: *L. reuteri* LTH2584 (■) versus LTH5448 (▲), TMW1.656 (●) and TMW1.112 (□). The three lineage I strains *L. reuteri* LTH2584, TMW1.656 and TMW1.112 could not be selectively enumerated by differential plate counts, therefore the sum of all three strains is shown in panels depicting place count data (Δ). Data are shown expressed as mean ± standard deviation from two independent experiments with two technique replicates in each experiment.

**Figure S4** pH value of sourdoughs fermented with binary or quaternary strain combinations after 1, 2, or 3 days of fermentation. Data are shown as mean ± standard deviation of all 20 samples taken from each strain combination. Values that do not share a common superscript differ significantly (p<0.05)

**Figure S5.** Total viable plate counts of sourdoughs fermented with binary or quaternary strain combinations after 1 day (black bars), 2 days (gray bars), or 3 days (white bars) of fermentation. Data are shown as mean ± standard deviation of all 20 samples taken from each strain combination. Values that do not share a common superscript differ significantly (p<0.05)
